# Molecular modeling and docking analysis of aspirin with pde7b in the context of neuro-inflammation

**DOI:** 10.6026/97320630016183

**Published:** 2020-02-29

**Authors:** Arthi Balasundaram, Darling Chellathai David

**Affiliations:** 1 Department of Pharmacology, Sri Ramachandra Medical College and Research Institute, Sri Ramachandra Institute of Higher Education and Research (DU), Porur, Chennai-116, Tamil Nadu, India

**Keywords:** aspirin, PDE7B, cognitive impairments, molecular docking, homology modelling

## Abstract

The PDE7B gene encodes 3'5'-cyclic nucleotide phosphodiesterase (PDE) and a known target in cognitive impairments. Therefore, it is of interest to design and development of potential inhibitors with PDE7B with improved binding features. We document that the amino acid residues such as H186, K190, and G113 of PDE7B protein showed crucial interactions with aspirin for further consideration in this context.

## Background

PDE7B enzyme is included under super-family of 11 members (PDE1-11), all involved in the hydrolyses of intracellular cyclic adenosine monophosphate (cAMP) and cyclic guanosine monophosphate (cGMP) in different cells. The regulating mechanisms of PDE genes expression are not well known [1]. Clare Gardiner et al, isolated human full-length cDNAs encoding a protein of 450 amino acids. This sequence of PDE7B showed highest homology (70% identity) to that of PDE7A [2]. J M Hetman et al, reported the full-length cDNA of PDE7B as 2399 bp, with 446 amino acids having a molecular mass of 50.1 kDa. This predicted protein sequences of PDE7B reveals an identity of 70% in the catalytic domain to that of PDE7. It was expressed in pancreas, brain, heart, thyroid, skeletal muscle, eye, ovary, submaxillary gland, epididymus, and liver [3]. T SASAKI et al, also reported the identification of PDE7B in caudate nucleus of human. The isolated cDNA showed 450 aminoacids with a molar mass of 51,835 Da. The expression of PDE7B was seen prominently in putamen and caudate nucleus of human brain [4].PDE7B overexpression showed tumor growth in glioblastoma animal model studies [5]. The phosphodiesterase7B inhibition is having a major role in regulating cognitive function through inhibition of cyclic AMP degradation. Increased levels of cAMP were shown to improve cognitive function and also provide neuroprotection [6].

Acetyl salicylic acid, commonly known as aspirin, is a known anti-inflammatory drug being used from almost 120 years. However, there is poor understanding of receptor pharmacology for this popular drug [7]. Aspirin is being used for primary and secondary prevention of cardiovascular diseases in both normal and diabetic patients. Many studies have reported poor benefits in primary prevention of cardiovascular diseases and positive association with increased risk of bleeding [8-10]. Also, benefits were reported with use of aspirin for the prevention of preeclampsia and intrauterine growth restriction [11]. Aspirin was tested against cognitive impairements in neurodegenerative diseases and ageing. [12,13] Though they have reported negative association of aspirin treatment and cognitive functions, the possibility for the effect was not revealed. Moreover, contradictory reports has been published related to this context [14]. Taking these in to consideration, a molecular docking study was conducted to evaluate the interactions of aspirin with PDE7B.

## Materials and Methods:

### Ligand and Protein preparation:

The drug molecule aspirin wasbretrieved from Pubchem database and loaded in the software ChemDraw Ultra version 12.0 to check the connection error in the bond order. The energy minimization was done by PRODRG Server [15]. The protein PDE7B (cAMP-specific 3',5'-cyclic phosphodiesterase 7B) is a crucial regulator of many critical physiological processes and didn't have the three-dimensional X-ray structure. This protein has a theoretical model which was old and removed from the PDB entries (1LXW) in 2002 (https://www.modelarchive.org/doi/10.5452/ma-ca9f6). Hence, a swiss model technique was used to develop a three dimensional modelled structure [16].The sequence of protein PDE7B (UniprotKB ID:Q9NP56) was retrieved and used to identify the template structure from the PDB source. The best-fit template (69.62%) for the protein sequence wasidentified using BLAST search and its PDB entry is 3G3N. After homology modelling, the best model was analysed based on the stereochemical quality of the model with PROCHECK analysis using the Ramachandran plot on SAVES server [17]. The possible ligand binding sites of the modelled target receptor was explored by Computer Atlas of Surface Topology of proteins (CASTp) server [18].

### Docking analysis:

The docking analysis was carried out using AutoDock Tools (ADT) v1.5.4 [19], including AutoDock and Autogrid v4.2 programs. The searching grid extended above the preferred target protein and the grid box was set as 80*80*80 in the XYZ angle; hydrogens were added to the ligandmoieties. Kollman charges were assigned and atomic salvation parameters were added to the protein atoms. Polar hydrogen charges of the Gasteiger-sort were allocated and the nonpolar hydrogens were combined with the carbons and the internal degrees of flexibility and torsions were set to the protein molecule. The compound aspirin wasdocked to target modelled protein PDE7B with the molecule considered as a rigid body and the ligands being flexible. Affinity maps for all the atom types present, as well as an electrostatic map, were computed with a grid spacing of 0.375Å. The search was completed with the Lamarckian Genetic Algorithm; populaces of 150 individuals with a modified rate of 0.02 were progressed for 10 eras. Evaluation of the results was done by sorting the different complexes concerning the predicted binding energy. ProteinsPlus retrieved the hydrophobic effect of the ligand by the help of PoseViewserver (http://proteinsplus.zbh.uni-hamburg.de/). All the images and protein-ligand interactions were visualized using PyMOL, (http://www.pymol.org) [20].

## Results and discussion:

**Figure 1:**
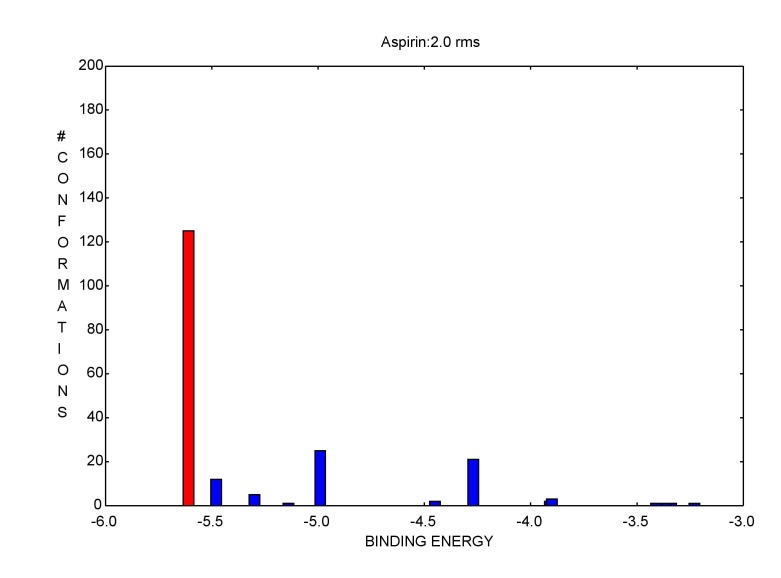
Binding energy and conformations of PDE7B. Red color indicates maximum number of conformations.

**Figure 2:**
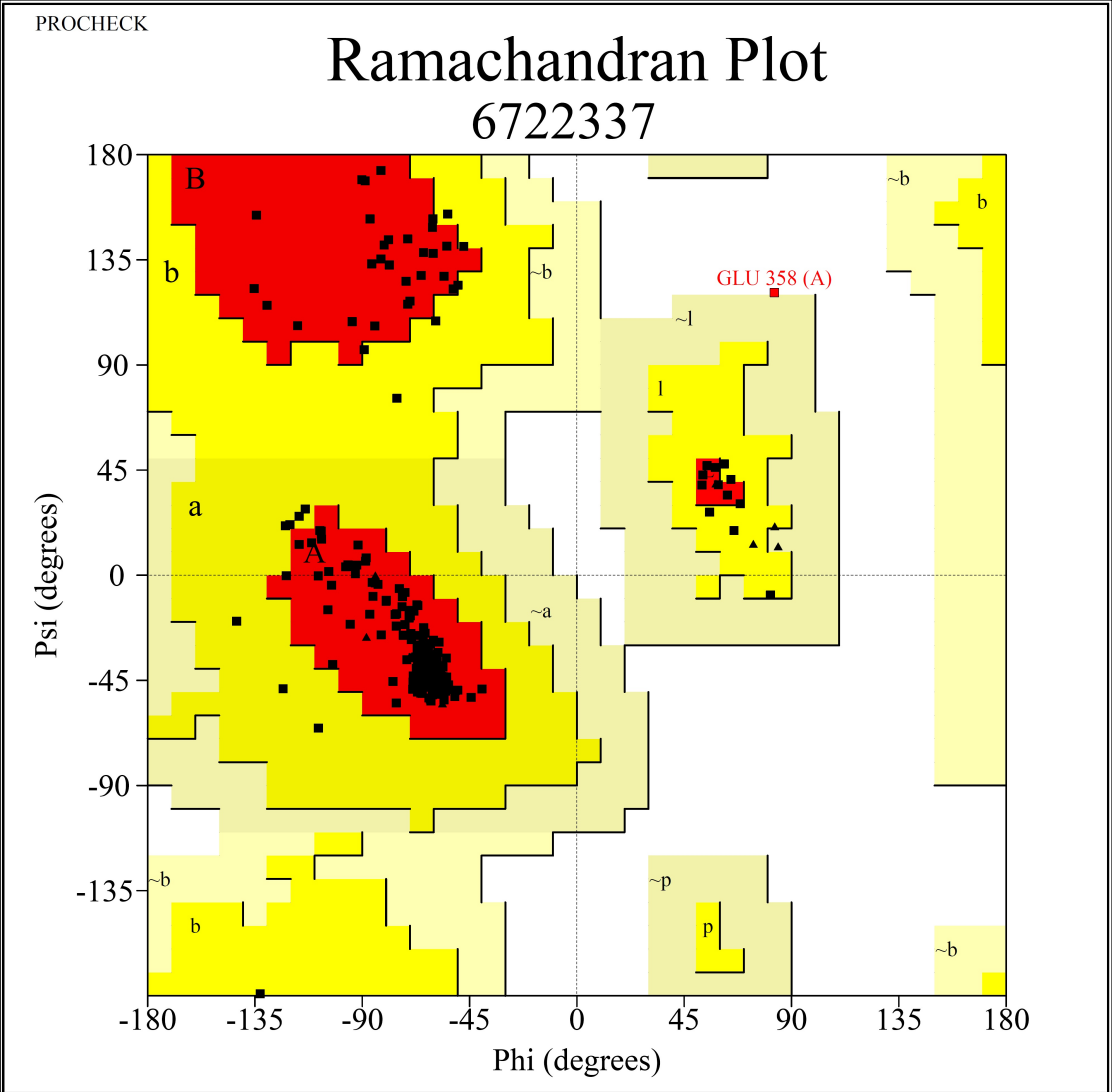
Protein structure prediction using Ramachandran Plot. [A,B,L]-residues in most favoured regions; [a,b,l,p]-residues in additional allowed regions; [∼a,∼b,∼l, ∼p]-residues in generously allowed regions

**Figure 3:**
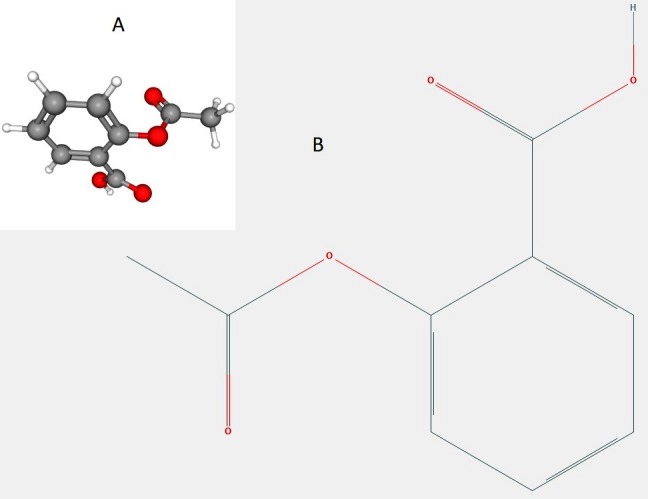
Chemical structure of aspirin, retrieved form PubChem. A- 3d view, B-2d view.

**Figure 4:**
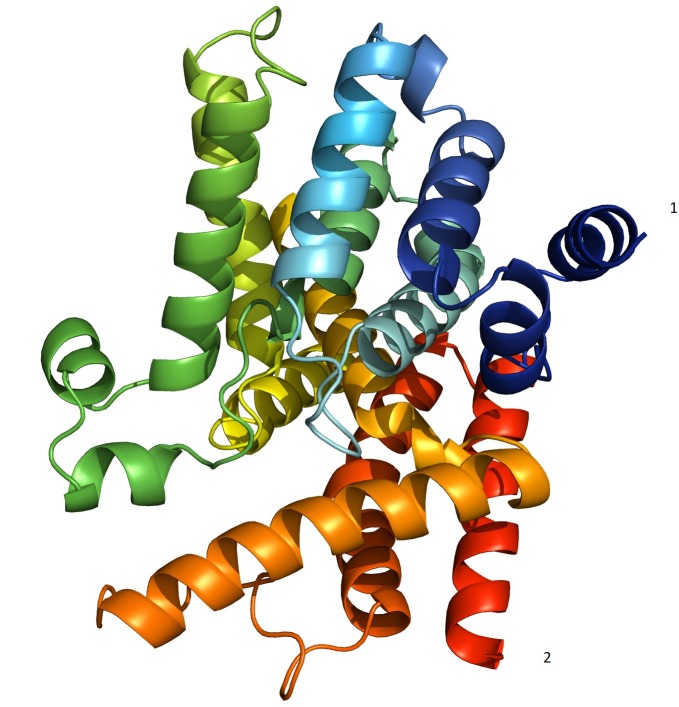
PDE7B structure obtained in homology modelling. 1-N terminus; 2-C terminus.

**Figure 5:**
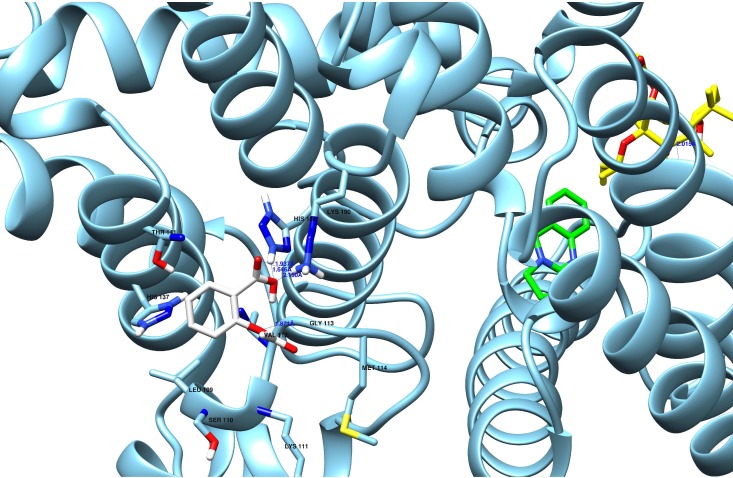
Molecular docking analysis of aspirin with PDE7B. Binding interactions were found with H186, K190, and G113 aminoacid residues.

Blind docking of Aspirin onto PDE7B was performed using AutoDock. The best docked conformation of the each of the ligands was identified through cluster analysis of 200 docked structures. The cluster with least binding energy and large number of conformations was chosen for the binding site analysis (Figure 1). The protein structure predicted using Ramachandran plot is depicted in (Figure 2). Based on Ramachandran plot analysis, 93.5% aminoacid residues were observed in favoured regions [A,B,L]. The structure of aspirin retrieved from PubChem is elucidated in (Figure 3). The protein structure obtained in homology modelling is described in (Figure 4). The best docked conformation of aspirin was located near the N-terminal region of the protein (Figure 5). The residues such as H186, K190, and G113 made crucial contacts with aspirin. The carboxylic group of aspirin formed two salt bridges with K190 (1.937 Å, 2.150 Å). It was also observed that H186 forming a stronger salt bridge with -COOH group of aspirin (1.646 Å). The Oxy group of acetyl formed a hydrogen bond with the backbone NH group of G113 (1.871). The aromatic ring of aspirin was in close proximity to V112. Unlike forskolin and vasicine, the interaction of aspirin was mainly modulated by salt bridges and hydrogen bonding rather than hydrophobic interactions. Also the binding site of Aspirin was ∼30 Å away from Forskolin/Vasicine's binding sites. The calculated binding energy was ∼5.61 KCal/mol (Table 1). Aspirin showed binding to H186, K190, and G113 amino acid residues of PDE7B. Previous studies have revealed that H173 is the active binding site of PDE7B protein which is located in the catalytic region of this protein. Contradictory results have been shown with aspirin and its effects on cognitive functions. Few studies have shown negative association between cognitive functions and aspirin treatment. A study by Matsumoto Chisa et al., (2017) has shown positive association with aspirin treatment and incidence of cognitive impairment [3]. The catalytic region of this protein lies between 172-410 amino acid residues. Vivek et al. (2012) have performed an in-silico study for designing potent inhibitors of PDE7B. The protein structure of PDE7B has been obtained from NCBI protein database and it's homology was found by BLASTp. Then model of target protein sequence was created by homology modelling. SAVS (PROCHECK) was used to analyse the models. Validation was done with Loop building and energy minimization, then best model acting as receptor was selected. LIGSITE was used to search the best pocket in the receptor where the inhibitor could bind. After running the LIGSITE, they revealed H173 as active site residue [21]. The interactions of vasicine and forskolin with PDE7B showed stronger binding affinity to PDE7B, which was reported by Balasundarm et al. (2015) [22].

## Conclusions:

We report the binding features of aspirin with the PDE7B enzyme in the context of cognitive impairments for further consideration.

## Figures and Tables

**Table 1:** Protein-Ligand docking scores

Ligand	Protein PDB ID	Binding amino acid Residues	Binding Energy (kcal/mol)	Inhibition Constant mM	vdW_HB desolv_energy (kcal/mol)	Ligand efficiency
Aspirin	PDE7B_HUMAN_cAMP_Modelled	His186, Lys190, Gly113	-5.61	1.72	-5.13	0.29
